# Validity and Reliability Family Adaptability and Cohesion Scales (FACES‐IV) in Peruvian Population

**DOI:** 10.1111/jmft.70185

**Published:** 2026-11-05

**Authors:** Anthony Copez‐Lonzoy, David Villarreal‐Zegarra, Nikol Mayo‐Puchoc, Willy Valle, Miguel Ipanaque, Salomón Huancahuire‐Vega, Lucy Puño‐Quispe, Julio Cjuno, Oscar Bazo‐Alvarez, Juan Carlos Bazo‐Alvarez

**Affiliations:** ^1^ Universidad San Ignacio de Loyola Lima Peru; ^2^ Instituto Peruano de Orientación Psicológica Lima Peru; ^3^ Universidad Científica del Sur Lima Peru; ^4^ Universidad Católica Los Ángeles de Chimbote Chimbote Peru; ^5^ Universidad Señor de Sipan Trujillo Peru; ^6^ Escuela de Psicología, Universidad Peruana Unión Juliaca Peru; ^7^ Escuela de Medicina Universidad San Juan Bautista Lima Peru; ^8^ Research Department of Primary Care & Population Health University College London London UK; ^9^ Escuela de Psicologia Universidad César Vallejo Trujillo Peru

**Keywords:** Circumplex Model, exploratory structural equation modeling, FACES‐IV, reliability, validity

## Abstract

This study aimed to (1) determine the psychometric properties and reliability of the FACES‐IV and (2) evaluate the curvilinear hypotheses of the Circumplex Model. A total of 6245 Peruvian participants, aged 18–65 years (*M* = 26.1; SD = 8.8), completed the Spanish version of the FACES‐IV. In addition, the complementary scales (PHQ‐9 and PSS) were administered for the evaluation of the Circumplex Model hypotheses. The six‐dimensional model, using an exploratory structural equation modeling (ESEM) approach, was robust. Reliability was acceptable for most dimensions (*ω* > 0.70). Balanced dimensions were associated with prevalence depression symptoms by 10% to 31% lower. Furthermore, the Rigid and Enmeshment dimensions increased family communication or satisfaction) by up to 35% to 94%. This study provides relevant information on the validity of the FACES‐IV when modeled using ESEM and provides psychotherapists and families with a psychometrically sound tool with clinical utility in the Spanish‐speaking population of Peru and similar countries.

## 1 Background

The assessment of family functioning plays a major role in clinical applications and research (Copez‐Lonzoy et al. 2016a; Olson 2011; Olson et al. 2019) since family interactions are often critical to the effectiveness of mental health treatments (Spitzer 1999). Family functioning is also a protective factor against the incidence of mental health problems such as depression and anxiety (Manea et al. 2015; Villarreal‐Zegarra et al. 2019). The use of psychometric tools for assisting the evaluation of family functioning has become widespread globally. A systematic review identified five main self‐administered family assessment instruments with strong psychometric properties that have been evaluated in 42 studies across five continents (Olson 2011). These scales have been applied in several research areas such as health, social sciences, and behavioral sciences (Olson 2011), as well as for the evaluation of community interventions (Wiegand‐Grefe et al. 2021) and clinical experiments (Henggeler and Schaeffer 2016). Although these scales are based on different theoretical models (e.g., McMaster model (Epstein et al. 1978) or Beavers Systems model (Beavers and Hampson 1990), one of these models has captured particular attention from researchers and shown to be useful in North and South American contexts (Beavers and Hampson 1990; Epstein et al. 1978; Olson 2000) the Circumplex Model by David Olson (Olson 2011; Olson 2000).

The Circumplex Model was conceived together with the Family Adaptability and Cohesion Evaluation Scales (FACES), a battery of tests for assessing different aspects of family functioning. The first version of FACES (Olson et al. 1979) emerged from a review of more than 200 concepts on family and couple relationships, which were grouped into three main dimensions (cohesion, adaptability, and communication) (Olson et al. 1979). Olson originally defined cohesion as “the emotional bonds that family members have with each other and the degree of personal autonomy they experience,” and adaptability as “the capacity of the family system to change its power structures, role relations, and relationship rules in response to situational and developmental stress” (Olson et al. 1979). In the Circumplex Model, cohesion and adaptability help determine balanced or unbalanced family functioning, while communication facilitates optimal functioning. The original FACES focused on the evaluation of cohesion and adaptability by using a set of 111 items (Larrosa 2002). In practice, this test was too long, and a shorter version was created, the FACES‐II.

The main purpose of FACES‐II was to provide a valid, reliable, and time‐efficient measure of family functioning in therapeutic contexts for assessing cohesion (16 items) and flexibility (14 items) that can evaluate the “real” (how the family actually is) and “ideal” (how the family would like to be) perceptions of family functioning (Larrosa 2002; Olson 2000). However, the FACES‐II version presented problems related to the “ideal” perception, so a separate scale was developed to assess family satisfaction (Schmidt et al. 2010). Furthermore, the adaptability dimension appeared to be particularly poorly defined and did not contribute to explaining family dysfunction (Beavers and Voeller 1983). Another important point was the failure to uphold the curvilinearity assumption of the Circumplex Model, which stated that very high or low levels of cohesion and adaptability are related to dysfunctional family relationships (Green et al. 1991). The orthogonality between the dimensions of cohesion and adaptability was not supported by the FACES‐II data, implying that both dimensions were closely related (i.e., non‐orthogonal factors), preventing a reliable classification of families into balanced and unbalanced functioning categories based on Olson's Circumplex Model (Olson 1985).

To overcome these limitations, the FACES III was developed and has been extensively studied in over 1200 studies. It includes a reduced version with 10 items for adaptability and 10 items for cohesion, as well as a communication scale (Kaslow 1996; Olson 1985). Although the FACES III proved more robust for clinical assessment, problems related to the orthogonality of the model were identified (e.g., the FACES‐III cohesion and adaptability scores showed a linear relationship with family dysfunction) (Barber and Buehler 1996; Crowley 1998; Olson 1985). This contradicts the curvilinearity of the Circumplex Model identified in other contexts, such as Latin America (Bazo‐Alvarez et al. 2016) and Europe (Schmidt et al. 2010; Tubiana‐Rufi et al. 1991) (i.e., the cohesion and adaptability dimensions of FACES‐III are not orthogonal). Furthermore, adaptability did not fit a model that was essentially unidimensional (Crowley 1998; Martínez Pampliega et al. 2006), impacting its reliability and prompting a major revision of its items (Kim and Park 2017). Additionally, the balanced adaptability measures of FACES‐III were not related to family well‐being, contradicting a key feature of the Circumplex Model (Barber and Buehler 1996).

FACES‐IV seeks to definitively overcome the limitations of its predecessors (Olson 2011). This version presents 42 items grouped into six dimensions, with seven items each. FACES‐IV incorporates two balanced dimensions (Balanced Cohesion and Balanced Flexibility) and four unbalanced dimensions (Rigid, Chaotic, Disengagement, and Enmeshment) (Olson 2011). The term “balanced” refers to the theoretical classification of adaptive versus dysfunctional functioning within Olson´s framework, rather than a hierarchical (bifactor models) or second‐order factor structure. Indeed, these dimensions do not represent a single linear continuum ranging from “functional” to “dysfunctional” family systems, but rather dynamics with opposing conceptual meanings. Adaptability was renamed “Flexibility” and redefined as “the amount of change in leadership, role relationships, and relationship rules” (Olson 2011; Olson and Gorall 2006). FACES‐IV addresses the curvilinearity problem in two steps. First, it develops six dimensions derived from the Circumplex Model within a unified multidimensional framework. Second, these scales are integrated into three ratios to measure how healthy or unbalanced the family functioning is (cohesion ratio, flexibility ratio, and total Circumplex ratio). In addition, the original study performed a discriminant analysis to identify balanced and unbalanced families, obtaining an average discrimination capacity of 78% (Olson 2011). Nevertheless, the issue of orthogonality among dimensions was not fully resolved in these versions.

As occurred with FACES‐III, cross‐cultural psychometric research on FACES‐IV has shown heterogeneous evidence of its psychometric properties (Baiocco et al. 2013; Gregorović Belaić et al. 2024; Costa Ball et al. 2013; Gomes et al. 2019; Vegas et al. 2022). Studies on Portuguese adolescents and adults (Gomes et al. 2019; Pereira and Teixeira 2013; Sequeira et al. 2021) agreed with Olson's FACES‐IV original structure. Conversely, studies in Uruguay, Italy, and Spain reported factorial structures with six dimensions but with a smaller number of items each (i.e., 24, 30, 36 items) (Costa Ball et al. 2013; Everri et al. 2020; Martínez Pampliega et al. 2006). This led to proposals for shorter versions of the original FACES‐IV (Arce‐Murillo et al. 2024; Gregorović Belaić et al. 2024; Everri et al. 2020; Martínez Pampliega et al. 2006; Newhart 2024a; Priest et al. 2020), including alternative factorial models to Olson's but keeping the main assumptions of the Circumplex Model; for example, using Exploratory Structural Equation Modeling (ESEM) (Everri et al. 2020). Given the evidence, we decided to contrast the factorial structure proposed by Olson against alternative ESEM solutions, which are detailed later in the methods section.

Although the original conceptualization of the Circumplex Model was developed within an Anglo‐Saxon framework prioritizing individual autonomy and clear personal boundaries, family dynamics in collectivist settings like Latin America reflect cultural values shaped by familism (e.g., high levels of emotional closeness, loyalty, or interdependence within the family structure) (Calzada et al. 2013; Schwartz et al. 2005; Shon et al. 2024; Smola and Fuligni 2024). In these cultural groups, relationships were observed between certain unbalanced dimensions (i.e., Enmeshment and Rigid) and balanced dimensions, wherein the former functioned as normative mechanisms for support and containment rather than as signs of family system dysfunction (Everri et al. 2020; Gomes et al. 2019; Vegas et al. 2022; Visani et al. 2025).

In a recent publication on the Circumplex Model (Olson et al. 2019), Olson strongly recommended researchers contrast key hypotheses from his model. In brief, one hypothesis says that balanced families are more functional (happy and well‐balanced) compared to unbalanced ones, while another hypothesis says that balanced families have more positive communication than unbalanced ones. The first hypothesis is connected with the curvilinearity assumption, and the second focused on the communication role. Since both hypotheses have been barely investigated, we contrasted them in our target population.

Furthermore, two core hypotheses regarding the predictive utility of the Circumplex Model deserve special attention. First, balanced families and couples experience higher levels of satisfaction than unbalanced ones; specifically, they may function as systemic protective factors (i.e., facilitating trust, emotional exchange, and adaptive coping) to mitigate severe stress and depressive symptoms (Olson 2011; Olson et al. 2019). Second, balanced families and couples exhibit more positive communication than unbalanced ones. This suggests that balanced dimensions and indices will be a protective factor for fluid family communication, while unbalanced family systems constitute a risk factor for both communication deficits and the maintenance of depressive symptoms (Olson et al. 2019).

In sum, we aimed to evaluate the psychometric properties of the FACES‐IV in the Peruvian young adult population. Our objectives were to (1) evaluate the FACES‐IV structural validity; (2) determine the reliability of the FACES‐IV scales; and (3) contrast two Circumplex Model hypotheses, considering the curvilinearity issue.

## 2 Method

### 2.1 Study Design

Psychometric study based on FACES‐IV cross‐sectional data collected in Peru between June and December 2020.

### 2.2 Participants and Data Collection

One family member (participant) completed FACES‐IV questions of survey. Participants were university students of both sexes, aged 18 to 65 years (*M* = 26.1; SD = 8.8), from public and private universities located in 27 Peruvian cities from three Peruvian regions (Coast, Jungle, and Highlands), who were parents or daughters/sons in their own families. Data collection was performed through a virtual survey that was distributed via official university channels (e.g, institutional e‐mails and online platforms) and promoted via selected social networks (i.e., Facebook and Twitter). Active promotion was sustained until enough data from the 3 Peruvian regions were collected. No missing data was identified in the evaluated sample.

### 2.3 Measurements

#### 2.3.1 Family Adaptability and Cohesion Evaluation Scale

The Family Adaptability and Cohesion Evaluation Scale (FACES‐IV) is made up of six dimensions of seven items each. Its design is Likert‐type, and its response options range from 1 (*completely disagree*) to 5 (*completely agree*). One set of 14 items measures two balanced dimensions (Balanced Cohesion and Balanced Flexibility). A second set of 28 items measured four unbalanced dimensions (Disengagement, Enmeshment, Rigid and Chaotic)(Olson 2011; Olson et al. 2019). The original FACES‐IV study reported valid and reliable measures for all dimensions (e.g., alpha: Balanced Cohesion = 0.89, and Balanced Flexibility = 0.80, Disengaged = 0.87, Enmeshment = 0.77, Rigid = 0.83, Chaotic = 0.85). In addition, Circumplex Ratio Scores were generated for the purpose of evaluating family behavior, Olson recommends using Cohesion and Flexibility Ratios to estimate the level of functional behaviour in the family system. These ratios can be calculated from the family cohesion and flexibility measures described above (Cohesion Ratio = Balanced Cohesion/(Disengaged + Enmeshment)/2; Flexibility Ratio = Balanced Flexibility/(Rigid + Chaotic)/2). The lower the ratio score below one, the more unbalanced the family functioning, and vice versa. The Total Circumplex Ratio is an average of the Cohesion and Flexibility Ratios (Olson 2011; Olson et al. 2019).

#### 2.3.2 Family Satisfaction

The Family Satisfaction Scale (FSS) is made up of 10 items (Likert‐type format) with response alternatives ranging from 1 (*very dissatisfied*) to 5 (*extremely satisfied*) [1,2]. The original scale showed optimal reliability (*α* = 0.92), adequate evidence for its unidimensional structure (CFI = 0.985; RMSEA = 0.048; SRMR = 0.043), and measurement invariance across men and woman (Koutra et al. 2013; Villarreal‐Zegarra et al. 2017).

#### 2.3.3 Family Communication

The Family Communication Scale (FCS) is also a 10‐item scale with an identical Likert‐type format and response interval (*completely disagree* (1) to *strongly agree* (5). In US people, the original study reported good reliability (*α* = 0.90) and validity (Koutra et al. 2013; Olson 2011). In Peru, an adaptation was performed with university students and showed similar validity (e.g., one‐dimensional, CFI > 0.95, RMSEA < 0.08) and reliability (*α* > 0.85) (Copez‐Lonzoy et al. 2016a).

#### 2.3.4 Depressive Symptoms

The Patient Health Questionnaire (PHQ‐9) is a scale based on nine depressive symptoms (DSM IV for major depression (MDD), which are assessed with four response options (0 = not at all; 1 = several days; 2 = more than half of the days; 3 = almost every day; total score from 0 to 27) (Spitzer 1999). In Peru, a one‐dimensional model was confirmed (CFI = 0.936; RMSEA = 0.089; SRMR = 0.039) with good reliability (*α* = 0.87 and *ω* = 0.87) (Villarreal‐Zegarra et al. 2019). A raw score ≥ 10 is interpreted as having moderate to severe depressive symptoms (Manea et al. 2015).

#### 2.3.5 Perceived Stress

The 10‐item version of the Perceived Stress Scale (PSS) questionnaire has two factors: (1) stress expression (6 items: 1, 2, 3, 8, 11, and 14) and (2) stress control (4 items: 6, 7, 9, and 10) (Lostaunau et al. 2017). This version was adapted for Spanish speakers, showing high reliability (*α* = 0.82) and a bi‐dimnesional structure (Remor 2006). We defined severe stress symptoms using a cut‐off of point ≥ 27 (Seedhom et al. 2019).

### 2.4 Covariates

Data was collected on sex (men/women), age (years), years of education, whether they have children (yes/no), civil status (married, divorced, widowed, cohabiting, separated, or single), and family‐income (“No income,” “Less than 1000 soles,” “Between 1000 to 1999 soles,” “Between 2000 to 3999 soles,” “Between 4000 to 5999 soles,” “Between 6000 to 9999 soles,” “Between 10,000 or more soles,” “I do not know”).

### 2.5 Statistical Analysis

#### 2.5.1 Internal Structure Validity

For the study variables, descriptive analyses were performed, reporting frequencies and percentages. To evaluate the internal structure of FACES IV, the confirmatory factor analysis (CFA) model was examined using the unweighted least squares estimator (WLSMV) (Brown 2015). For model evaluation, the comparative fit index (CFI) was used; for parsimony, the root mean square error of approximation (RMSEA) ≤ 0.06 was used, and additionally, the root mean square of standardized residuals ≤ 0.08 (SRMR). In CFA models, items with negligible factor loadings (< 0.30) (Brown 2015) or those presented as reverse‐scored items will be removed (Kline 2005). Furthermore, other fit indices for comparing nested models were implemented using maximum likelihood estimation (MLM), such as the Akaike Information Criterion (AIC) and the Bayesian Information Criterion (BIC), which considers low values as optimal as a better measure for model comparison (Brown 2015). Furthermore, an exploratory structural equation modeling (ESEM) approach was implemented in the most plausible models, allowing for flexibility in rotation (i.e., exploratory factor analysis) and robustness in the fit indices (CFA) (Marsh et al. 2020). This is because the FACES indicators (items) are designed to capture the complexity of family behavior and do not necessarily present “perfect” indicators, but rather can be dimensionally complex items (i.e., a specific indicator can contribute to different dimensions). This strategy has demonstrated better results in obtaining the “less inflated factor” relationship, improving the discriminant validity of the construct, which can more realistically approximate the data (Marsh et al. 2014). Target rotation was used as a semi‐confirmatory oblique method that allows for the free estimation of cross‐loadings (i.e., it does not force them to zero) and the reduction of differential bias (e.g., reducing the overestimation of latent factors) (Marsh et al. 2014, 2020), making it ideal for complex multidimensional models (e.g., FACES‐IV). We analyzed the original model based on the history of high correlation between its factors and the complexity of some items (Martínez‐Pampliega et al. 2017; Newhart 2024a; Sebokova et al. 2016; Vegas et al. 2022).

The first model (M1) was based on a correlated six‐factor (CF) model (Arce‐Murillo et al. 2024; Desautels et al. 2016; Olson 2011; Türkdoğan et al. 2018; Vegas et al. 2022). The second model (M2) evaluated the performance of M1 using an ESEM strategy. The third model (M3) eliminated potentially negative or non‐functional items (11); the fourth model (M4) was based on the ESEM model of version M3 (i.e., without reversed or irrelevant items). In addition, two alternative models were evaluated on the Flexibility (M5) and Cohesion (M6) scales. Sample size determination was informed by recent simulation research on multidimensional ESEM models, which indicates that large sample sizes (*N* > 4000) are necessary for adequate parameter recovery (Garrido et al. 2020). Based on these guidelines, we conducted a Monte Carlo simulation study to evaluate parameter estimation and factor congruence (mean c.c >0.95). The results confirmed that a final sample size of *N* = 6245 was sufficient to achieve excellent recovery and exceed the standard congruence threshold (see Supporting Information S1: Table S1).

#### 2.5.2 Reliability

For the reliability of the scores, the alpha (*α*) and McDonald's omega (*ω*) (Flora 2020) reliability coefficients were used. The latter shows greater precision in the absence of the equivalence‐tau and other assumptions used for the observable variables. Values above 0.70 were considered optimal for both coefficients. In addition, the average inter‐item correlation was evaluated as a measure of indicator cohesion for brief scales (e.g., thresholds 15 to 0.40) (Clark and Watson 2019).

#### 2.5.3 Circumplex Model Hypotheses

The hypotheses of the circumplex model are based on the relationship between balanced/unbalanced families and family functionality. We define balanced families as those where their individuals do not present mental health problems such as depressive symptoms (PHQ ≥ 10 points) or perception of severe stress (PSS ≥ 27 points). In addition, a balanced family is one in which its members perceive high family satisfaction and high family communication (top tercile). Therefore, the outcomes were depressive symptoms, severe stress, family satisfaction, and family communication (outcomes were analyzed independently). Our exposure variables were the dimensions Balanced Cohesion, Balanced Flexibility, Disengagement, Enmeshment, Rigid, Chaotic, and Ratios. The covariates considered were sex (male/female), age group, whether you have children (yes/no), whether your family strictly complies with quarantine (yes/no), whether you received an economic bonus (yes/no), and whether your family earns less than 2000 per month (yes/no). Because exposure was in different units of measurement, these variables were transformed into z‐scores to facilitate their interpretation. Univariate and multivariable (adjusted) Poisson regression models were fit to evaluate the differences between the different hypotheses posed by the circumplex model.

#### 2.5.4 Ethics Topics

The research team was responsible for safeguarding the rights, safety, and well‐being of the participants. Also, the data was anonymous and confidential. This study has been approved by the ethics committee of the Instituto Peruano de Orientación Psicológica (CIEI‐IPOPS‐22).

## 3 Results

### 3.1 Participants

The data collection collected 6245 participants who met the inclusion criteria of our study (included participants). The largest proportion of included participants were female (*n* = 3985, 63.8%), had no children (*n* = 4372, 70.0%), single (*n* = 4730, 75.7%), and only 27.9% reported family income above the minimum wage (*n* = 1744). The mean age of the participants was 26.2 years (SD = 8.8). The sociodemographic characteristics of the participants is detailed in Table 1.

**Table 1:** Characteristic of the study participants (*n* = 6245).

	*n*	%
Sex		
Women	3985	63.80
Men	2260	36.20
With children		
No	4372	70.00
Yes	1873	30.00
Civil status		
Married	616	9.90
Divorced	55	0.90
Widowed	29	0.50
Cohabiting	652	10.40
Separated	163	2.60
Single	4730	75.70
Age group		
16 to 19	1440	23.10
20 to 29	3211	51.40
30 to 39	998	16.00
40 to 49	428	6.90
50 to 65	168	2.70
Family income		
No income	1,559	25.00
Less than 280 dollars	1,842	29.50
280 to 560 dollars	999	16.00
560 to 1120 dollars	491	7.90
1120 to 1680 dollars	142	2.30
1680 to 2800 dollars	70	1.10
2800 or more dollars	42	0.70
I do not know	1,100	17.60

*Note:* During the evaluation, the minimum wage in Peru was 260 US dollar.

Abbreviations: *n*, number of participants; %, percentage.

### 3.2 Internal Structure Validity

Initially, the original six‐factor model (M1) was evaluated and showed a poor fit. Therefore, this original model was re‐evaluated with the removal of these negative items showed items with negative loadings on the Enmeshment dimension (4, 10, 16, 22, 28, 34) and the Rigid dimension (5, 23, 29), and were therefore excluded from the analyses to generate a new model (M3), which showed a significantly better fit compared to the original model. However, both models (M1 and M3) exhibited problems with excessively high correlations between the dimensions of the FACES IV (*r* > 0.95). To mitigate these potentially overestimated relationships between the dimensions and to achieve a more realistic level of shared variability among the indicators, two ESEM models were implemented. The first ESEM model (M2), based on the original structure, achieved optimal fit indices (CFI = 0.975; TLI = 0.966; SRMR = 0.016; RMSEA = 0.039; AIC = 715002.92; BIC = 717166.309), while the second ESEM model, based on the version without negative items (M4), achieved a superior fit (CFI = 0.983; TLI = 0.974; SRMR = 0.014; RMSEA = 0.040; AIC = 550273.74; BIC = 551951.884). Both ESEM models (M2 and M4) demonstrated improved model fit and more realistic correlations between factors (*r* < 0.57) (see Table 2; Figures 1 and 2). The target loadings from the ESEM model revealed that the balanced dimensions share substantial variance with one another. The dimension with the greatest structural prominence was the Chaotic dimension, with loadings reaching up to 0.649 (Item CH24). Certain indicators of Enmeshment and Rigid showed conceptual overlap with Balanced Cohesion and Balanced Flexibility, consistent with expressions of familism characterized by control and over‐involvement in family dynamics (Table 3).

**Table 2:** Confirmatory factor analysis and exploratory structural equation modeling for FACES IV model (*n* = 6245).

model	*x* ^2^ (*df*)	CFI	TLI	SRMR	RMSEA	AIC	BIC
M1	69,235.169 (804)	0.718	0.689	0.123	0.117	729,194.22	729,861.429
M2	6657.592 (624)	0.975	0.966	0.016	0.039	715,002.92	717,166.309
M3*	13,953.904 (480)	0.923	0.916	0.055	0.072	554,017.9	554,557.058
M4	3834.116 (345)	0.983	0.974	0.014	0.040	550,273.74	551,951.884
M5	1322.423 (102)	0.943	0.933	0.050	0.044	281,438.2	281,667.341
M6	1189.152 (116)	0.959	0.952	0.036	0.044	286,486.32	286,735.687

Abbreviations: *X* ^2^, chi‐squared; CFI, comparative fit index; *df*, degrees of freedom; M1, original FACES IV model; M2, ESEM based original model; M3, original model without negative items; M4, ESEM based M3; M5, cohesion scale without negative item; M6, flexibility scales without negative item; RMSEA, root mean square error of approximation; SRMR, standardized root mean square residual; TLI, Tucker–Lewis index.

* Negative items deleted = 10,16,22,34,40,5,11,23,29,41.

**Figure 1:**
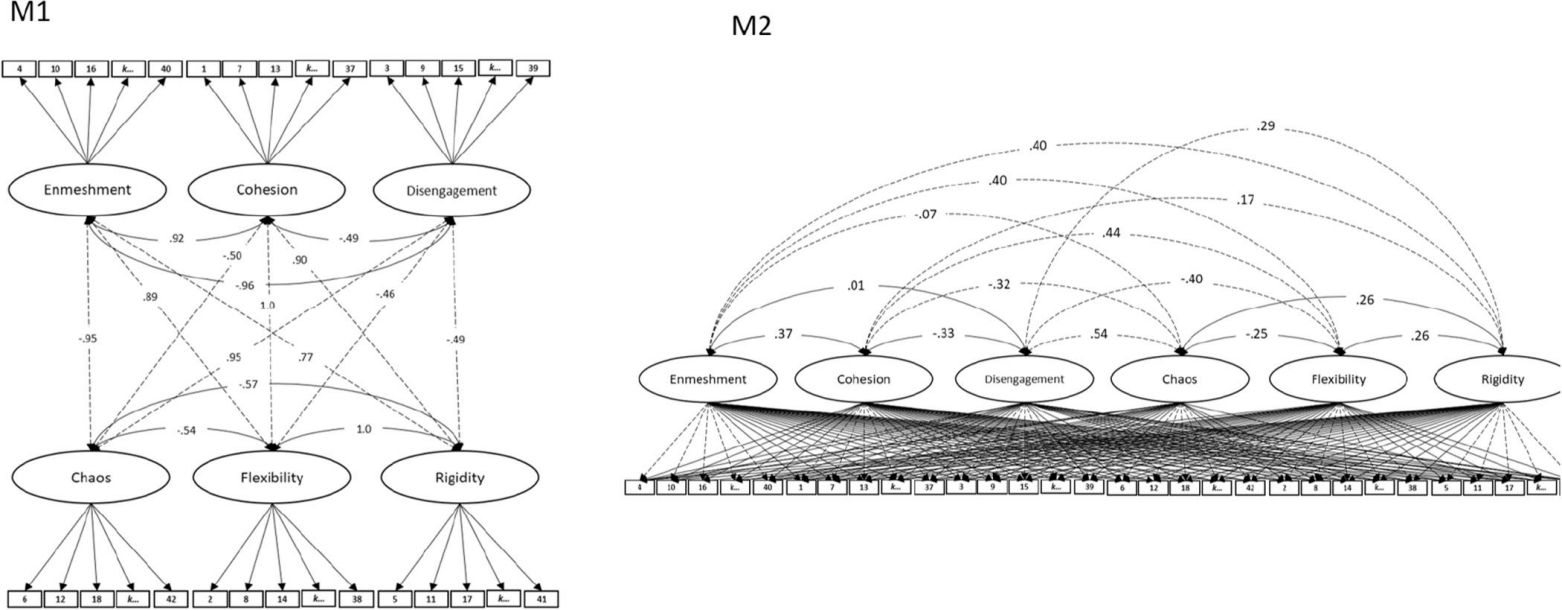
Graphical representation of the different types of models evaluated in this study. Confirmatory factor analysis (M1) and Exploratory structural equations modeling (M2) of the original Family Adaptability and Cohesion Evaluation Scale. Solid lines represent correlations within the cohesion or flexibility dimensions, while dotted lines represent correlations across all dimensions.

**Figure 2:**
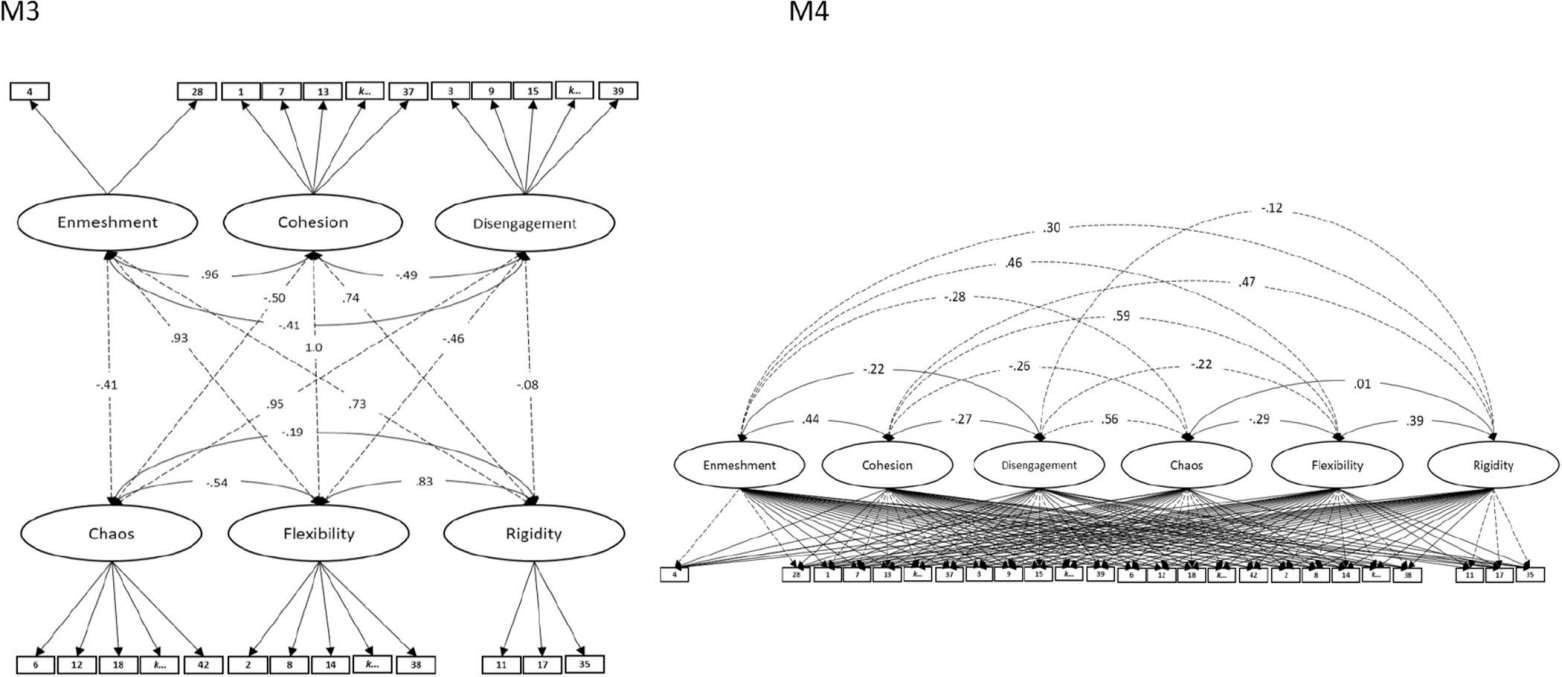
Graphical representation of the different types of models evaluated in this study. Confirmatory factor analysis (M3) and Exploratory structural equations modeling (M4) of the modified Family Adaptability and Cohesion Evaluation Scale. Solid lines represent correlations within the cohesion or flexibility dimensions, while dotted lines represent correlations across all dimensions.

**Table 3:** Factor loadings of ESEM model.

Items	Enmeshment	Cohesion	Disengaged	Chaotic	Flexibility	Rigid
EM4	0.443***	0.093***	−0.02	0.113***	0.254***	0.008
EM28	0.293***	0.416***	−0.188***	0.114***	0.102***	0.146***
CO1	0.139***	0.466***	0.381***	−0.200***	0.135*	−0.328***
CO7	0.361***	0.239***	−0.116***	0.042**	0.390***	−0.080***
CO13	0.135***	0.203***	−0.150***	−0.050**	0.545***	0.061**
CO19	0.015	0.355***	−0.149***	−0.045**	0.384***	0.136***
CO25	0.331***	0.424***	−0.260***	0.202***	0.009	0.154***
CO31	0.009	0.527***	−0.096***	0.050**	0.124***	0.055**
CO37	−0.090***	0.450***	0.004	−0.006	0.127***	0.251***
DIS3	0.132***	0.057*	0.588***	0.083*	−0.155***	0.049*
DIS9	0.057**	−0.116***	0.435***	0.188***	−0.074***	0.297***
DIS15	0.007	−0.036	0.192***	0.293***	0.145***	0.194***
DIS21	−0.139***	0.094***	0.372***	0.358***	0.009**	0.014
DIS27	−0.213***	−0.149***	0.331***	0.394***	0.106***	0.073***
DIS33	−0.096***	−0.175***	0.289***	0.499***	−0.002	0.128***
DIS39	−0.209***	0.016	0.344***	0.440***	−0.014**	−0.02
CH6	−0.03	0.024	0.422***	0.319***	−0.102***	−0.084***
CH12	0.157***	−0.008	0.234***	0.505***	−0.140***	0.046**
CH18	−0.003	0.013	0.299***	0.437***	−0.048**	−0.027*
CH24	0.026	−0.037*	0.152***	0.649***	0.021	−0.075***
CH30	0.059***	−0.037	0.138***	0.631***	−0.151***	−0.040**
CH36	−0.164***	0.073***	0.182***	0.522***	0.084***	−0.0043**
CH42	−0.104***	−0.038*	0.281***	0.498***	−0.066***	−0.076***
FLE2	0.211***	0.239***	0.119*	−0.125***	0.432***	−0.034*
FLE8	0.261***	0.127***	−0.034	−0.058***	0.419***	0.082***
FLE14	0.150***	0.052***	−0.074***	−0.084***	0.470***	0.333***
FLE20	−0.078***	0.443***	−0.102***	−0.053**	0.406***	0.091***
FLE26	0.227***	0.320***	−0.140***	−0.025	0.036	0.327***
FLE32	0.115***	0.299***	0.041*	−0.213***	0.090***	0.499***
FLE38	−0.015	0.505***	−0.103***	−0.074***	0.214***	0.154***
RI11	0.014**	0.008	0.281***	−0.095***	0.249***	0.309***
RI17	0.193***	−0.064***	0.181***	−0.051**	0.211***	0.564***
RI35	−0.014**	0.146***	0.120***	−0.150***	0.144***	0.608***

* *p* < 0.05

** *p* < 0.1

*** *p* < 0.001.

### 3.3 Reliability

The Balanced cohesion, Balanced flexibility, Disengaged, and Chaotic dimensions were found to have adequate levels of internal consistency (>0.70). However, the Enmeshment (items EM4 and EM8) and Rigid (items RI11, RI17, and RI35) dimensions presented low levels of internal consistency. However, the average inter‐item correlation was acceptable for Enmeshment (AIIC = 0.39) and Rigid (AIIC = 0.34). In addition, the general dimensions of cohesion and flexibility presented adequate levels of internal consistency (see Table 4).

**Table 4:** Reliability values of FACES‐IV (*n* = 6245).

	*α*	*ω*	Mean	SD
Balanced cohesion	0.78	0.78	26.90	4.84
Balanced flexibility	0.86	0.86	27.96	5.14
Disengaged	0.79	0.79	17.49	5.76
Enmeshement	0.63	0.65	20.85	4.26
Rigid	0.66	0.68	21.92	4.73
Chaotic	0.83	0.83	15.99	5.92

Abbreviations: *α*, alpha coefficient; *ω*, omega coefficient.

### 3.4 Circumplex Model Hypotheses

#### 3.4.1 Hypothesis 1

The adjusted models identified that balanced dimensions and ratios were inversely associated with depressive symptoms, being linked to a 10% to 31% lower prevalence. Conversely, unbalanced dimensions were significantly associated with a higher prevalence of depressive symptoms (38% to 45% higher) and severe stress (up to 53% higher) across most dimensions. Enmeshment and Rigid dimensions presented a divergent pattern, being associated with a 16% to 20% lower prevalence of depressive symptoms and a 40% to 94% higher prevalence of family satisfaction. Furthermore, balanced ratios and dimensions were associated with a more than 2.4‐fold higher prevalence of family satisfaction (*p* < 0.05). Our findings partially support the hypothesis that depressive symptoms and severe stress are less prevalent among individuals with higher scores in balanced dimensions of the FACES‐IV model (see Table 5).

**Table 5:** Regression model between family functionality and outcomes of balanced families.

Scale and ratio	Depressive symptoms^a^	Perceived stress^a^	Satisfaction family^a^	Communication family^b^
	PR adjusted*	PR adjusted*	PR adjusted*	PR adjusted*
Balanced cohesion	**0.75 [0.68–0.82]**	1.16 [0.97–1.98]	**2.41 [2.11–2.74]**	**2.21 [1.97–2.49]**
Balanced flexibility	**0.73 [0.66–0.80]**	1.12 [0.94–1.13]	**2.82 [2.47–3.30]**	**2.42 [2.13–2.74]**
Disengaged	**1.38 [1.25–1.53]**	**1.53 [1.29–1.81]**	**0.62 [0.56–0.69]**	**0.68 [0.61–0.75]**
Enmeshement	**0.80 [0.72–0.88]**	1.18 [0.99–1.40]	**1.94 [1.71–2.20]**	**1.85 [1.65–2.07]**
Rigidity	**0.84 [0.76–0.93]**	**1.28 [1.07–1.54]**	**1.40 [1.27–1.55]**	**1.35 [1.23–1.47]**
Chaotic	**1.45 [1.31–1.60]**	**1.51 [1.28–1.77]**	**0.61 [0.64–0.68]**	**0.65 [0.59–0.72]**
Cohesion Ratio	**0.69 [0.62–0.76]**	0.95 [0.80–1.12]	**2.42 [2.14–2.72]**	**2.18 [1.96–2.43]**
Flexibility Ratio	**0.90 [0.81–0.99]**	**1.46 [1.23–1.75]**	**1.59 [1.44–1.76]**	**1.52 [1.38–1.66]**
Total Circumplex Ratio	**0.74 [0.67–0.81]**	1.14 [0.95–1.35]	**2.78 [2.48–3.18]**	**2.43 [2.16–2.74]**

*Note:* Values in bold were significant (*p* < 0.05).

^a^ Outcomes correspond to hypothesis 1.

^b^ Outcomes correspond to hypothesis 2. Adjusted model by sex (men/women), age (years), years of education, whether they have children, civil status, and family income.

#### 3.4.2 Hypothesis 2

Our study identified that balanced dimensions and ratios were associated with a higher prevalence of high levels of family communication (up to 2.4 times higher). In addition, unbalanced dimensions (e.g., Disengaged and Chaotic) were associated with a 35% lower prevalence of family communication. These findings are consistent with the second hypothesis of the Circumplex Models (see Table 4).

## 4 Discussion

### 4.1 Main Findings

The FACES IV was validated in a Peruvian population, testing two hypotheses of the circumflex model (Olson et al. 2019). The assessment of the internal structure maintained all the dimensions originally proposed by Olson (Olson 2011; Olson et al. 2019) using an exploratory structural equation model (ESEM). However, the Enmeshed and Rigid dimensions showed problematic indicators (e.g., lower and negative factor loadings), which led to a version with fewer items. The hypotheses tested showed that perceived stress was positively related to the balanced dimensions (i.e., Cohesion and Flexibility). Similarly, the Enmeshed and Rigid dimensions were directly associated with family communication by almost twofold, which could imply that Peruvian families perceive these dimensions as factors that contribute to the maintenance of positive family ties.

### 4.2 Comparison With Other Studies

#### 4.2.1 Dimensionality

The six‐factor model composed of the two balanced scales (Cohesion and Flexibility) and four unbalanced scales (Rigid, Enmeshment, Chaotic, and Disengaged) of the FACES‐IV was replicated in a Peruvian population. However, the evaluation of the original model proposed by Olson under a traditional approach (i.e., confirmatory factor analysis) showed insufficient fit and inverse indicators identified in previous studies (Vegas et al. 2022; Visani et al. 2025). Furthermore, the CFA model showed extremely strong correlations between factors regardless of their membership (i.e., intra‐ and inter‐dimensional relationships), which could imply a conceptual overlap between factors (Gregorović Belaić et al. 2024; Newhart 2024a). However, other studies showed that the FACES‐IV had an acceptable evaluation, but with significant changes in its dimensions due to possible cultural characteristics or specific age groups (Baiocco et al. 2013; Gregorović Belaić et al. 2024; Newhart 2024b). This could lead to a choice to merge factors or the artificial generation of a general factor that might lose its discriminatory power in individual assessments (Gregorović Belaić et al. 2024; Newhart 2024b).

For this reason, our study demonstrated that the measurement of constructs that address the complexity of family systems (e.g., FACES‐IV) must acknowledge that items rarely have an isolated, unidimensional meaning (i.e., indicators restricted only to their own dimension) (Marsh et al. 2014; Vail et al. 2025). Under the exploratory structural equation modeling (ESEM) framework, indicators have the potential to provide information shared between items (i.e., free cross‐loadings) capturing the shared systemic variance, characteristic of family dynamics (Marsh et al. 2014), as is the case in the assessment of family systems (Everri et al. 2020; Vail et al. 2025). Indeed, by accounting for cross‐loadings, ESEM mitigates the potential overestimation of correlations between factors typical in traditional CFA models, yielding more realistic values similar to those found in other studies (Everri et al. 2020). Likewise, specific indicators of Enmeshment and Rigid showed conceptual overlap, primarily with the balanced dimensions. For example, item EM28 reflected high emotional closeness, whereas item RI11 intertwines normative structures (Flexibility) with a strong continuum of familism.

This analytical approach involves two important conditions: first, up to 35% of the variance is shared by the dimensions (e.g., cohesion and flexibility balanced) condition expected in the assessment of family systems; second, more than 60% of the variance of the dimensions remains completely independent (i.e., consistent with Olson's curvilinear model). In this way, the clear separation rules out the possibility of overlap or merging of dimensions identified in prior studies (Gregorović Belaić et al. 2024; Desautels et al. 2016; Newhart 2024b). Consequently, the items retain the ability to discriminate between affective closeness (Cohesion) and the ability to follow roles and rules (Flexibility) comprehensive tool that helps couples and families identify their current situation and track their progress (Wojciak and Olson 2025). Indeed, this establishes that FACES‐IV is a valid instrument for the Peruvian population.

### 4.3 Reliability

Reliability was acceptable across most dimensions of the FACES‐IV model, consistent with previous findings (Koutra et al. 2013; Olson 2011; Pereira and Teixeira 2013; Vegas et al. 2022). In particular, the balanced Cohesion and Flexibility scales achieved higher reliability scores than those of other cultural groups (Koutra et al. 2013; Olson 2011; Priest et al. 2020). Conversely, the unbalanced scales obtained lower reliability scores than the balanced ones (e.g., Rigid and Enmeshment). This pattern can be attributed to three main factors: a) cultural differences in the perception of family closeness, where items intended to capture pathology (e.g., “Family members feel pressured to spend most free time together”) are often interpreted positively in collectivist contexts characterized by strong familism, b) the reduced item count following model optimization (two items for Enmeshment and three for Rigid); and c) heterogeneity of observed reliability in international adaptations (Arce‐Murillo et al. 2024; Gregorović Belaić et al. 2024; Costa Ball et al. 2013; Desautels et al. 2016; Everri et al. 2020; Martínez‐Pampliega et al. 2017; Pereira and Teixeira 2013; Priest et al. 2020; Sarour and El Keshky 2022; Sequeira et al. 2021; Türkdoğan et al. 2018; Vegas et al. 2022; Visani et al. 2025), consistent with our findings. Because conventional reliability coefficients are sensitive to scale length, the average inter‐item correlation (AIIC) was evaluated. The AIIC values for Enmeshment and Rigid fell well within acceptable thresholds (0.15 to 0.50), confirming adequate internal cohesion among the remaining indicators are despite the abbreviated scale length (Clark and Watson 2019). However, readers should interpret the findings regarding both scales with caution. The results underscore the need to re‐evaluate the indicators of Enmeshment and Rigid in Peruvian or Latin American populations in general, in order to identify standardized items that capture cultural diversity and can distinguish functional family solidarity from the clinical dysfunction that is central to the assessment of family systems in these cultural contexts.

### 4.4 Circumplex Model Hypotheses

Our study partially refined the hypotheses of the Circumplex model by confirming that balanced families tend to demonstrate higher functionality and communication, and that the dimensions of Enmeshment and Rigid may present an unexpectedly favorable profile in this population. The Circumplex Model suggests that unbalanced dimensions (i.e., Enmeshment, Rigid, Chaos, and Disconnection) are associated with adverse family dynamics, such as (a) greater emotional distress due to less effective conflict resolution strategies through support and interaction among family members, and (b) lower levels of resilience linked to reduced emotional well‐being (Oshri et al. 2015). Our findings revealed discordant conditions in some unbalanced dimensions (i.e., Enmeshment and Rigid) regarding variables that facilitate cohesion and flexibility.

This discrepancy is not exclusive to our context, as it has been reported in Mediterranean countries (Spain, Portugal, Italy, and Greece) (Vegas 2025). These cultural groups may perceive that high levels of closeness and rigidity are not entirely negative (Vegas 2025). Instead, these traits may function as regulatory mechanisms, associated with lower emotional symptoms (e.g., depression or stress) and an enhanced perception of belonging, thereby being linked to lower family stress and higher self‐esteem and communication (Hernández and Bámaca‐Colbert 2016). These dimensions are coherently linked to familism (i.e., a distinctive feature of Latin and Mediterranean societies) by considering a solid, collaborative, and trust‐based family relationship as an important aspect of their lives, rather than as extreme closeness or rigidity (Ma et al. 2017; Schwartz et al. 2005; Shon et al. 2024). Furthermore, closeness and family ties are especially important for these family groups; consequently, increasing the bonds of emotional and psychological well‐being (Gámez‐Guadix and Almendros 2015; Vegas 2025). In addition, behavioral rigidity is not necessarily perceived as a negative dynamic in collectivist societies that tend to promote control and discipline, unlike in English‐speaking countries (Vegas 2025).

Our findings suggest that the Cohesion Ratio is associated with an emotional containment framework that preserves closeness among members without exceeding the limits toward harmful enmeshment. Regarding the Flexibility Ratio, the results highlight the “paradox of systemic adaptation.” While in non‐clinical samples this ratio reflects everyday resilience, in clinical populations (such as cancer patients), flexibility takes on a more demanding dimension: the need to constantly modify routines, norms, and financial responsibilities (i.e., often assumed by primary caregivers) implies a high cognitive load. Thus, achieving “balanced” flexibility requires intense systemic negotiation. Taken together, these indicators suggest that a balanced family system is inversely associated with emotional distress (e.g., depressive symptoms), being characteristic of families that effectively regulate collective emotions even in the face of the demands of illness.

### 4.5 Implications for Research and Practice

The confirmation of the FACES‐IV six‐factor model for assessing family functioning has important implications for clinical practice and psychological assessment. The FACES‐IV allows for a broader understanding of family dynamics. In clinical settings, the Circumplex Model can be assessed through the subscales to identify current family dynamics and relevant problems in family functioning (Olson 2011; Olson et al. 2019). Furthermore, professionals can use the subscales as a tool to expand their knowledge of possible parenting styles, which may be useful in the initial identification of family members with extreme dynamics (e.g., chaotic or disengaged) (Olson et al. 2019). This assessment also allows for the identification of developmental changes in therapy sessions, which can help professionals design strategies aimed at fostering positive family dynamics as part of the evolution during treatment.

In the Latin American context, the Enmeshment and Rigid subscales require a particular interpretation, given their association with a positive perception related to the degree of family satisfaction and communication. Therefore, professionals working in collectivist environments characterized by high familism (e.g., Mediterranean countries) (Hernández and Bámaca‐Colbert 2016) should bear in mind that high scores on these dimensions do not necessarily imply pathological behaviors (Vegas 2025). On the contrary, they may indicate a strong and reliable family relationship based on closeness and family ties perceived as important. Furthermore, this would broaden the understanding of family bonding patterns, potentially opening a window of plausible possibilities for designing culturally adapted intervention strategies aimed at channeling affective closeness and emotional dependence within the family (see Figure 3).

**Figure 3:**
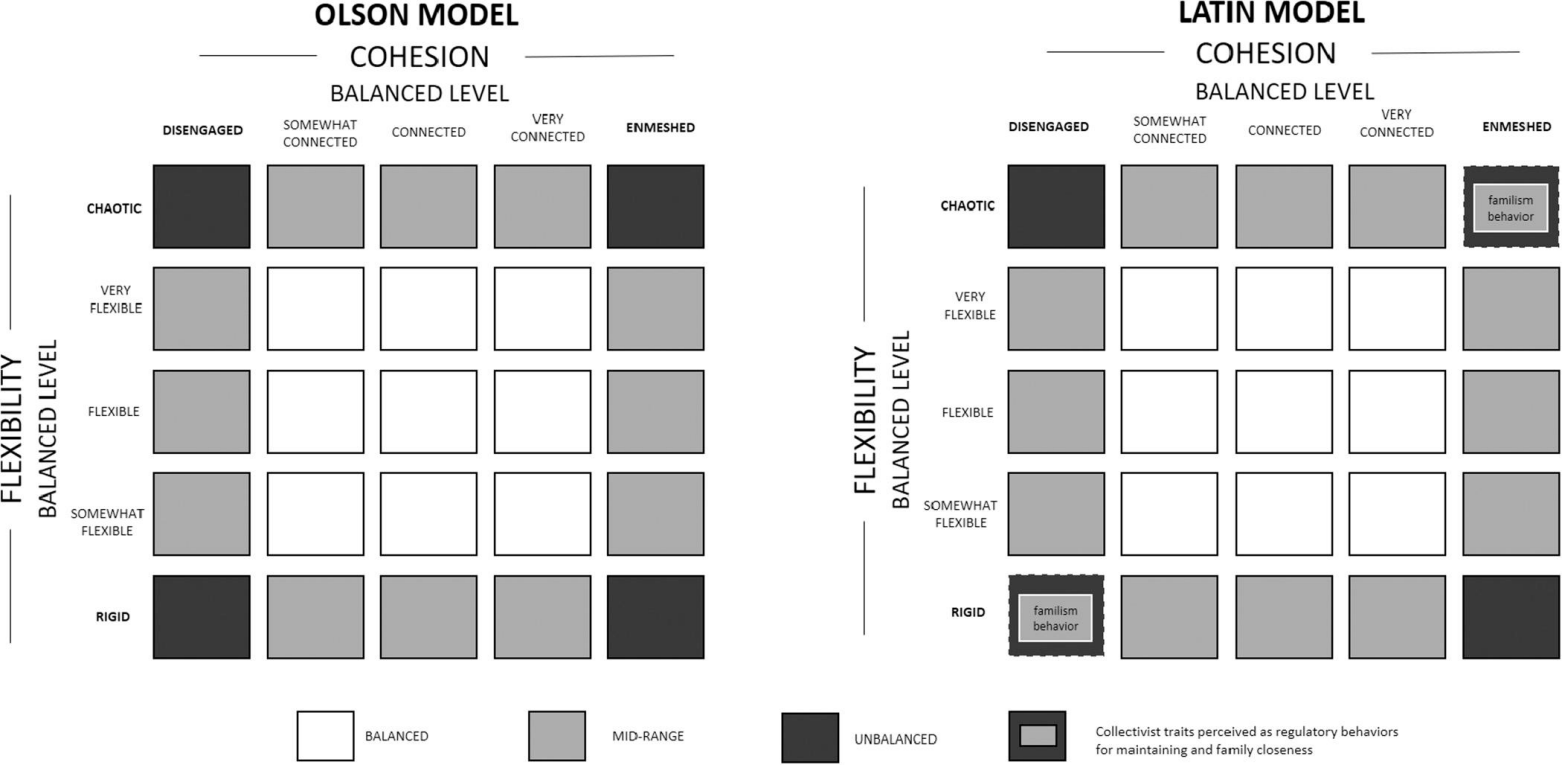
Comparison between Olson's model and Peruvian findings.

From the researchers’ standpoint, the six‐factor model of FACES‐IV maintains conceptual clarity in the evaluation of family models. Due to the relevance of family environments to health, the Circumplex Model allows for the exploration of the relationship between different dimensions and clinically relevant components in family dynamics (e.g., assessment of codependency in substance addictions, gender‐based violence, behaviors related to primary caregivers in families dealing with or caregivers of patients with chronic non‐communicable diseases, etc.) (Ahmed and Earl 2019; Marsac and Alderfer 2011; Wahab et al. 2021). Therefore, FACES‐IV provides clinically relevant measures that can be useful in large‐scale population assessments, contributing to a better understanding of family functioning and its influence on health throughout the lifespan (Priest et al. 2020).

## 5 Strengths and Limitations

We identified several strengths and limitations in our study. A key strength is that this is the first large‐scale study to evaluate the psychometric structure of the full version of the FACES‐IV in a Latin American context. Our study directly addresses the international call made by Olson regarding the functioning of the FACES‐IV model in diverse cultural groups (e.g., the Peruvian population) based on the functioning of balanced and unbalanced families. Our findings provide crucial cross‐cultural evidence of the functioning of the FACES‐IV subscales. In particular, dimensions theoretically considered negative (i.e., Enmeshment and Rigid) function as adaptive components in Peruvian family regulation, thus expanding the theoretical boundaries of the model. Regarding limitations, data were collected from a single family member, rather than from entire families. While single‐informant assessment is common practice in psychometric evaluations involving family variables (i.e., Olson's original study), it is important to note that our approach captures the individual perception of family dynamics rather than a multi‐informant systemic assessment. To mitigate the potential overestimation of variance inherent to single‐informant designs, we integrated key complementary criteria, such as structural information, health indicators, and family composition. Furthermore, other components of family dynamics (e.g., role differences or problem‐solving) were not assessed, which does not directly compromise the validity of our main findings. Therefore, we proactively addressed these limitations by integrating mental health indicators (i.e., symptoms of anxiety, depression, or severe stress) and systemic components (e.g., communication and family satisfaction). Despite these limitations, our study provides reliable and culturally relevant evidence that can contribute to Circumplex Model hypotheses on a large scale in the Peruvian population.

## 6 Conclusions

Our study identified that the ESEM model consistently replicates the original six‐dimensional model in the general population of Peru. Most dimensions obtained acceptable reliability scores on the FACES‐IV model. Our findings confirm the protective role of balanced relationships within the Circumplex Model and reveal cultural divergence among the unbalanced dimensions. In the Peruvian context, Rigid and Enmeshment do not function strictly as maladaptive traits, but rather as potentially adaptive components of family functioning, suggesting the evolutionary measurement of family dynamics based on a collectivist family framework of the Peruvian population.

## Supporting information


Supporting File

## Data Availability

The data that support the findings of this study are available from the corresponding author upon reasonable request.
