# Supplementary material for: Validity and Reliability Family Adaptability and Cohesion Scales (FACES‐IV) in Peruvian Population
Source: J Marital Fam Ther. 2026 Nov 5;53(1):e70185. doi: 10.1111/jmft.70185 (PMC13736818; doi:10.1111/jmft.70185)
Supplement: Supplementary file 1 [file JMFT-53-0-s001.docx]

Supplemental Material

TS1

Mean factor congruence between the optimal population structure (Monte Carlo Simulation)

| Factors | Items | c.c. mean |
| --- | --- | --- |
| Factor 1 | x1,x7,x13,x19,x25,x31,x37 | .99 |
| Factor 2 | x2,x8,x14,x20,x26,x32,x38 | .99 |
| Factor 3 | x3,x9,x15,x21,x27,x33,x39 | .99 |
| Factor 4 | x4,x10,x16,x22,x28,x34,x40 | .99 |
| Factor 5 | x5,x11,x17,x23,x29,x35,x41 | .99 |
| Factor 6 | x6,x12,x18,x24,x30,x36,x42 | .99 |

Note. Congruence coefficients higher than .95 (N = 4000)
